# Investigation of associated factors with post-operative outcomes in patients undergoing Tetralogy of Fallot correction

**DOI:** 10.1186/s12893-018-0338-y

**Published:** 2018-03-15

**Authors:** Ahmad Ali Amirghofran, Jamshid Badr, Mansour Jannati

**Affiliations:** 1 Associated Professor, Shiraz University of Medical Sciences, Shiraz, Iran; 2 Cardiovascular Surgeon, Shiraz University of Medical Sciences, Shiraz, Iran; 3 Department of Cardiovascular surgery, Faghihi Hospital, Shiraz University of Medical Sciences, Shiraz, Iran

**Keywords:** Tetralogy of Fallot, Mortality, ICU Stay, Extubation, Haematocrit

## Abstract

**Background:**

Tetralogy of Fallot (TOF) is one of the congenital cardiac abnormality which occurs during embryonic time. Although surgical correction, especially early operation, is the best way to treat patients, still contributing factors in morbidity and mortality is controversial. The objective of this study is to investigate potential factors which might be correlated with post-operative outcomes of TOF.

**Methods:**

In this retrospective study, 349 monitored patients with TOF correction were selected. Median of age was 4 (0.66–8) year, 58% of patients were male and 42% were female. Time of inotropic drug, extubation time, and ICU stay were considered as post-operative outcomes which extension of each of them increased the risk of death.

**Results:**

Ventricular septal defect enlargement was associated with longer extubation time and ICU stay. Higher ratio of pre-operative haematocrit was correlated with mortality (0.047).

**Conclusions:**

Results of this study demonstrated that haematocrit ratio before operation should be considered as a predictive factor, and patients with higher ratio of haematocrit require more care after operation. VSD enlargement is associated with longer extubation time and ICU stay.

## Background

Tetralogy of Fallot (TOF) is a cardiac abnormality that occurs during embryogenesis [1, 2]. TOF has been known as one of the most common causes of cyanotic heart disease during first ages of life with probability of occurs of 3 in every 10,000 live births which approximately include 7%–10% of all congenital defects [3]. The risk of death in the first year of life is very high, after the first year is constant until age 25 years, and then increases [4]. Four distinct anatomic features which characterize TOF include: (1) overriding aortic root, (2) pulmonary outflow tract obstruction, (3) ventricular septal defect (VSD), and (4) right ventricular hypertrophy (which occurs as result of the obstruction to pulmonary blood flow) [5].TOF is a multifactorial disease and its etiology has been associated with various factors such as maternal intake of retinoic acid, untreated maternal diabetes and chromosomal anomalies [3]. Also, JAG1 mutations in Alagille syndrome has been associated with TOF [6] Surgically untreated patients are in danger of the death due to hypoxic spells (main cause), cerebrovascular accidents, and brain abscesses [7].

During last century, surgical methods have developed to correct TOF [5]. The early attempts at correction of TOF were followed with high rate of mortality in young children that propelled surgeons to perform surgical treatments in two steps: a shunt operation within the first 2 or 3 years followed by complete cardiopulmonary bypass correction in older ages [8]. The ideal age for correction of children with cyanotic TOF has recently been a controversial topic. These argument totally consider the rate of morbidity and mortality regarding various ages of operation and the correlation between early operation and normal development of the heart and lungs after correction [9–13]. A number of evidences demonstrated that early TOF correction reduces the side effects on vital organs, including the heart itself which can suffer from hypoxemia, and enhances survival and physiological result [13–15]. Generally, the rate of morbidity and mortality after operation has been considered to be reduced by increasing the size of patient [10, 13], however, TOF correction in the first year of life can support medical benefits, economic and psycho-social advantages [16].

Early correction of TOF can be along with trans- and post-operative matters; for example, complete repair in young age requires utilization of transannular patching (TAP) [17, 18]. Likely, increase in the need for a transannular patching might compromise right ventricular function in the late postoperative period [11]. On the other hand, implanting a transannular patch can be a significant risk factor for children with a body surface area less than 0.48 m^2^ [12]. Moreover, the type of repair can impress the right ventricle outflow tract and its function in the late postoperative period [19, 20]. The late one is particularly important in the cases of correction in the first year of life, because of more frequent use of transannular patches in this group of patients.

Regarding absence of consensus about the best time of correction and the best type of repair, the objective of this study is to investigate the effective pre-, tans-, and post-operative factors with respect to mortality and morbidity outcomes in TOF patients that can be used as helpful surgical pre-considerations.

## Methods

### Patients

This retrospective study was performed on 382 patients with Tetralogy of Fallot who undertook correction surgery from 2001 to 2010 in three heart centres in Shiraz.

### Inclusion and exclusion criteria

Thirty three patients who had either of homograft utilization, or re-operation, or only BT shunt (Blalock–Thomas–Taussig shunt) were removed from study and the rest 349 with complete correction were entered into study.

### Performance

Information of each patient was recorded on separate form for statistical analysis as following: Demographic information including age, gender, weight, HCT, type of surgery, previous surgery. Trans-operative information including pump time, Ventricular Septal Defect (VSD) enlargement, transannular patching, monocusp utilization, PA/RV gradient, operation approach. Post-operative information including inotrope time, extubation time, length of ICU stay, and post-operative side effects including heart block, bleeding and death.

### Ethical consideration

The study was carried out in accordance with the Declaration of Helsinki, and the ethics committee of the Shiraz University of Medical Sciences approved the protocols of the study. The patients’ records were kept confidential.

### Data analysis

Qualitative and quantitative data were reported as frequency (%) and median (range), respectively. Statistical analysis was performed using Chi-square, t-test, multiple regression and logistic regression, and *P* value < 0.05 was considered significant. All data were analyzed with IBM SPSS Statistics for Windows, version 18.0 (SPSS Inc., Chicago, Illinois, USA).

## Results

Of 382 patients, those homograft was used for them, or underwent redo surgery, or just had BT Shunt Surgery were excluded and demographic information of 349 patients with tetralogy of fallot on whom complete correction was performed has been presented in Table 1. This information include pre-, trans-, and post-operative characteristics of patients under study.

**Table 1:** Pre-, trans-, and post-operative characteristics of patients^a^

Variable	Value
Pre-operative	
Age, year	4 (0.66–8)
Gender	
Male	204 (58)
Female	145 (42)
Weight, Kg	6 (4–7)
Previous operation	90 (25.8)
Haematocrit (HCT), %	48 (11.5–77.8)
Trans-operative	
Pump time, min	79 (24–280)
VSD Enlargement	133 (38.1)
Transannular patching	158 (45.3)
Pulmonary monocusp	40 (11.5)
Transatrial Myectomy	77 (22.1)
RV-LV Ratio	60 (10–137)
RV-PA-Gradiant, mmHg	20 (0–84)
Post-operative	
Time of inotropic drug, hrs	46 (0–360)
Extubation time, hrs	8 (0–211)
ICU-stay, day	3 (1–69)
Bleeding to operation	17 (4.9)
Heart block	11 (3.2)
Post operation mortality	15 (4.3)

^a^Data are presented as Median (range) and No. (%)

Statistical analysis in order to find any correlation between post-operative and pre/trans operative variables showed that previous operation reduce the length of ICU stay and extubation time. Also, occurrence of VSD enlargement is in direct relation with inotrope time, ICU stay, and extubation time. Moreover, the increasing length of inotrope time increases the mortality risk. In a same way, increasing length of extubation time increases the risk of bleeding (Table 2).

**Table 2:** Correlation of post-operative variables with pre- and trans-operative ones^a^

	Time of inotropic drug			ICU stay			Extubation time		
	Yes	No	*P*-value	Yes	No	*P*-value	Yes	No	*P*-value
Previous Operation	49.3 ± 27.7	53.54 ± 36.5	0.307	3.4 ± 2.2	4.8 ± 7.9	0.013	8.2 ± 6.4	14.7 ± 25.6	≤ 0.001
VSD Enlargement	57.34 ± 49.27	49.67 ± 20.45	0.098	5.5 ± 7.7	3.8 ± 6.4	0.033	17.5 ± 32	10.5 ± 11.5	0.024
Heart block	47.44 ± 20.6	52.7 ± 34.9	0.652	3.5 ± 1.9	4.5 ± 7.1		15.3 ± 14.6	13.1 ± 22.8	0.676
Pulmonary-monocusp	66.85 ± 74.42	50.33 ± 25.62	0.21	–	–	–	–	–	–
Transatrial Myectomy	48.2 ± 22.6	53.6 ± 37.22	0.12	–	–	–	–	–	–
Transannular Patch (TAP)				4.7 ± 6.7	4.3 ± 7.3	0.606	15.5 ± 30.2	11.3 ± 12.8	0.113
Bleeding				5.2 ± 2.2	4.4 ± 7.1	0.681	46 ± 77.3	11.8 ± 16.6	0.014
Dead	70 ± 97.16	52 ± 31.8	0.014	–	–	–	102.4 ± 100	11.7 ± 16.2	≤ 0.001

^a^Data are presented as Mean ± standard deviation

In order to make a more accurate knowledge about the correlation between post-operative and pre/trans operative variables, regression analysis was performed on data (Table 3). The results showed that there is a correlation inotrope time and dead and Pump time. Pump time, RV-LV Ratio, bleeding, and dead have correlation with Extubation time.

**Table 3:** Regression analysis between post-operative independent variables and pre/trans-operative variables^a^

	Time of inotropic drug^b^		Extubation time^b^		ICU-stay^b^		Death^c^	
	Β coefficient	*P*-value	Β coefficient	*P*-value	Β coefficient	*P*-value	Β coefficient	*P*-value
Age	0.072	0.198	−0.142	0.011	− 0.099	0.072	− 0.047	0.377
Previous operation	−0.061	0.399	−0.079	0.12	−0.005	0.943	−0.133	0.93
HCT	0.019	0.795	−0.069	0.172	−0.179	0.021	0.096	0.182
Transatrial myectomy	0.031	0.680	−0.029	0.581	0.117	0.14		–
pulmonary monocusp	0.046	0.582	−0.065	0.325	0.021	0.807		–
Pump time	0.18	0.015	0.259	≤ 0.001	0.072	0.358		–
VSD Enlargement	0.008	0.907	0.003	0.946	0.069	0.355	0.084	0.959
Transannular patching	−0.037	0.675	0.046	0.448	−0.023	0.8	−0.052	0.596
RV-LV Ratio	0.109	0.208	0.118	0.05	0.101	0.268	0.044	0.339
RV-LV Grad	−0.065	0.446	–	–	–	–		–
Heart block	−0.053	0.433	0.020	0.68	−0.025	0.73	−2.77	0.212
bleeding	0.091	0.252	0.138	0.009	−0.005	0.952	−4.8	0.003
Dead	0.332	≤ 0.001	0.65	≤ 0.001	0.172	0.028		–

^a^Data are presented as regression *P*-value between two distinct variable

^b^Multiple regression analysis

^c^Logistic regression analysis

As presented in Table 4, there is a statistical significant relation between the pump time and mortality; the longer pump time, and the higher probability of death. Likewise, the higher percentage of HCT significantly increase the risk of mortality.

**Table 4:** Mortality dependence to some independent variables^a^

	Dead	Alive	*P*-Value
Age	4.9 ± 5.5	6.5 ± 6.9	0.37
Pump-time, hr	119 ± 67.7	80.8 ± 25.2	0.047
RV-LV Ratio	64.7 ± 9.8	62 ± 18.6	0.363
HCT, %	59.9 ± 10.5	48.6 ± 10.5	0.047

^a^Data are presented as Mean ± standard deviation

## Discussion

Tetrallogy of fallot is the oldest surgically treated congenital heart disease which is well documented due to many studies have been done around it in long-term [21]. Nonetheless, post-operative morbidity and mortality are of main concerns in regard with TOF surgically treatment. Recently, nuclear magnetic resonance has being valuably contributed to evaluate cardiac function to postoperative follow-ups of patients with tetralogy of Fallot [22] and predicts exercise capacity in adult operated tetralogy of Fallot [23]. In this regard, older age at operation, previous heart failure, and high > 0.5 RV:LV systolic pressure are considered predictors of late mortality [24]. In this study, we investigated other potential factors which might correlate with post-operative outcomes.

Early correction of TOF has been suggested as the best solution, as it decrease the rate of morbidity and mortality and low incidence of transannular patching [25]. According to our results the age of patients who died were less than whom survived; however this difference was not significant. Monocusp valves replacement has been reported to operate effectively in the early postoperative period, although there are concerns about the long-term efficiency of monocusp. Totally, no significant correlation was observed between monocusp pulmonary replacement and post-operative outcomes of correction [26–28]. Similarly, in the present study, pulmonary monocusp replacement was not associated with postoperative outcomes. In a study conducted on 80 patient in Pakistan, there was no significant statistical relation between heart block and different types of patching, however, it was demonstrated that transannular patching trigger to bleeding and as result reoperation of patients (*P* = 0.001) [29]. In our study, on the other hand, heart block was not significantly correlated with post-operative outcomes.

The transatrial and transatrial-transpulmonary approach is frequently used today in all age groups, including neonates, with survival rate close to 100% and a low incidence of early reintervention [25, 30, 31]. Stewart et al. [32] reviewed 102 patients (median age 5.9 months) comparing transatrial approaches with TAP. They found that postoperative RV outflow tract gradients were greater in the transatrial and transatrial-transpulmonary groups than in the Transannular patching group. In present study, no correlation was found between approach of operation and neither of ventricular outflow or morbidity outcomes.

Logistic regression analysis showed that bleeding incidence correlates with death outcome. Also, According to mortality analysis of present study, duration of pump time in dead patients was significantly longer compare with survived patients. Likewise, the percentage of HCT before surgery in cases with death outcome was higher than those survived (0.047). In a same way, Guevara et al. showed that preoperative haematocrit showed statistically significant association with 30-day mortality [33]. Actually haematocrit increases in TOF patients as a response to hypoxia [34]. However, in another study it was demonstrated that the haematocrit level did not correlate with mortality [35].

Regarding the ICU stay outcome, patient who had carried out previous operation required shorter ICU stay. In other words, previous operation shorten the length of ICU stay. Also, increase of VSD enlargement significantly increased the length of ICU stay. Lee et al. demonstrated that patients who underwent early repair for TOF showed more VSD closure and longer ICU stay, although they did not indicate the correlation between these two factors [36]. Moreover, multiple regression analysis demonstrated that haematocrit ratio indirectly and dead outcome directly correlate with length of ICU stay. Similar to our findings, Benbrik eat al. demonstrated that patient with higher level of haematocrit undertook shorter period of ICU stay [35]. Based on our results, increasing the length of inotrope time was correlated with dead outcome. This correlation was also confirmed by multiple linear regression analysis (*P* < 0.001). However, it has been shown that patients with less time of inotropic drug exhibited similar mortality rate compare with patients who underwent longer time of inotropic drug [35].

The average of extubation time among patients with previous operation background was more than those without previous experience. Also, incidence of VSD enlargement and bleeding were significantly in relation with increase of extubation time. However, some studies have not shown any relation between extubation time and VSD [37]. In addition, those patients with dead outcome has showed longer extubation time. The results of multiple regression also demonstrated that extubation time directly correlates with pump time and RV-LV ratio, along with bleeding and dead. Although our results indicated to the significance of RV-LV ratio, other studies did not show RV-LV ratio as a significant factor which can be different in relation with different variables [38].

## Conclusion

In conclusion, results of this study demonstrated that haematocrit ratio before operation should be considered as a predictive factor, and patients with higher ratio of haematocrit require more care after operation. VSD enlargement is associated with longer extubation time and ICU stay. Also, longer time of inotropic drug, extubation time, and ICU stay are threatening and alerting the incidence of death. Thus, extension of each of these factors should be paid more attention.

## Abbreviations

HCT: Hematocrit
ICU: Intensive care unit
LV: Left ventricular
RV: Right ventricular
TAP: Transannular patch
TOF: Tetralogy of fallot
VSD: Ventricular septal defect
